# BilR is a gut microbial enzyme that reduces bilirubin to urobilinogen

**DOI:** 10.1038/s41564-023-01549-x

**Published:** 2024-01-03

**Authors:** Brantley Hall, Sophia Levy, Keith Dufault-Thompson, Gabriela Arp, Aoshu Zhong, Glory Minabou Ndjite, Ashley Weiss, Domenick Braccia, Conor Jenkins, Maggie R. Grant, Stephenie Abeysinghe, Yiyan Yang, Madison D. Jermain, Chih Hao Wu, Bing Ma, Xiaofang Jiang

**Affiliations:** 1 Department of Cell Biology and Molecular Genetics, University of Maryland, College Park, College Park, MD USA; 2 Center for Bioinformatics and Computational Biology, University of Maryland, College Park, College Park, MD USA; 3 National Library of Medicine, National Institutes of Health, Bethesda, MD USA; 4 Division of Molecular and Cellular Biology, Eunice Kennedy Shriver National Institute of Child Health and Human Development, Bethesda, MD USA; 5 Department of Chemistry and Biochemistry, University of Maryland, College Park, MD USA; 6 Program of Computational Biology, Bioinformatics, and Genomics, University of Maryland, College Park, MD USA; 7 Institute for Genome Sciences, Department of Microbiology and Immunology, University of Maryland School of Medicine, Baltimore, MD USA

**Keywords:** Bacterial genes, Gastrointestinal diseases, Metagenomics

## Abstract

Metabolism of haem by-products such as bilirubin by humans and their gut microbiota is essential to human health, as excess serum bilirubin can cause jaundice and even neurological damage. The bacterial enzymes that reduce bilirubin to urobilinogen, a key step in this pathway, have remained unidentified. Here we used biochemical analyses and comparative genomics to identify BilR as a gut-microbiota-derived bilirubin reductase that reduces bilirubin to urobilinogen. We delineated the BilR sequences from similar reductases through the identification of key residues critical for bilirubin reduction and found that BilR is predominantly encoded by Firmicutes species. Analysis of human gut metagenomes revealed that BilR is nearly ubiquitous in healthy adults, but prevalence is decreased in neonates and individuals with inflammatory bowel disease. This discovery sheds light on the role of the gut microbiome in bilirubin metabolism and highlights the significance of the gut–liver axis in maintaining bilirubin homeostasis.

## Abstract (web summary)

BilR is identified as a bilirubin reductase encoded by gut bacteria, which reduces bilirubin to urobilinogen.

## Main

**Fig. 1:**
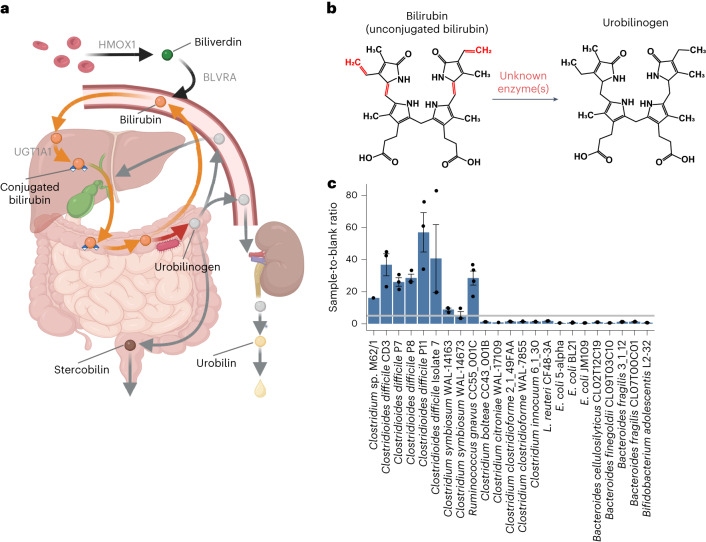
Identification of bilirubin-reducing bacterial strains. **a**, Illustrated representation of the haem degradation pathway. Key human enzymes are labelled with grey text. **b**, Diagram of the structures of bilirubin and urobilinogen. The bonds reduced during bilirubin reduction are shown in red. **c**, Results of fluorescence assay screening of bacterial strains. Measurements from *n* = 3 independent biological replicates are shown as black points. Bars show the ratios of the samples’ fluorescence to a corresponding abiotic media sample with bilirubin added. Error bars indicate 1 s.e. above and below the mean values. The grey line marks a ratio of 5, above which the sample was considered to be positive for bilirubin reduction. *Clostridium* sp. M62/1 and *Clostridium citroniae* WAL-17108 are represented by single data points.

Bilirubin, an intermediate of the haem degradation pathway, plays a critical role in human physiology through the gut–liver axis^1^. Alongside other organic molecules in bile such as cholesterol and bile acids, bilirubin diglucuronide (conjugated bilirubin) is secreted into the gut where it is either excreted or reabsorbed. When bilirubin diglucuronide is deconjugated by human or bacterial beta-glucuronidases into unconjugated bilirubin, it can be readily reabsorbed into the enterohepatic circulation or further metabolized via reduction reactions by gut microorganisms into the more excretable metabolites urobilinogen and stercobilinogen^2^. Bilirubin reabsorption elevates serum bilirubin levels, while excretion as urobilinogen and stercobilinogen in stool and urine facilitates its clearance, completing the haem degradation pathway^3–5^ (Fig. 1a).

Dysregulation of gut microbial bilirubin reduction affects serum bilirubin levels, which can have substantial health implications. In moderate concentrations, bilirubin serves as an important antioxidant with potential health benefits^6,7^. However, elevated serum bilirubin concentrations can become toxic, leading to jaundice and, in extreme cases, kernicterus, a type of bilirubin-induced neurological damage^8^. Similarly, urobilinogen can be reabsorbed and has been associated with multiple diseases, highlighting the key role of bilirubin reduction in the homeostasis of multiple metabolites^3–5,9,10^. While the importance of bilirubin enterohepatic circulation to serum bilirubin levels was suggested in the 1960s and 1970s, it was largely undervalued until studies in the early 2000s showed the direct impact of microbial bilirubin metabolism on serum bilirubin levels in rats^11–14^.

Gut microorganisms are solely responsible for the reduction of bilirubin to urobilinogen, potentially using the molecule as a terminal electron acceptor in anaerobic respiration. Despite the recognized role of gut microorganisms in bilirubin reduction, the gut microbial enzyme that reduces bilirubin to urobilinogen, hereafter called bilirubin reductase, has remained undiscovered^15^. Multiple bilirubin-reducing bacteria have been identified, including strains of *Clostridioides difficile*^12^, *Clostridium ramosum*^16^, *Clostridium perfringens*^12^ and *Bacteroides fragilis*^17^, but much of this work was performed before genome sequencing was widely available, making it difficult to know which strains were studied. Without knowledge of the gene encoding bilirubin reductase, it is difficult to draw conclusions about how gut microbial metabolism affects serum bilirubin homeostasis, leaving an important gap in our understanding of the haem degradation pathway.

In this study, we aimed to identify the enzyme(s) responsible for bilirubin reduction to urobilinogen and apply this knowledge to understand the relationship between microbial bilirubin reduction and human health. Combining experimental screening of common gut bacteria, comparative genomics and biochemical inference, we were able to identify a candidate bilirubin reductase gene we named *bilR* and confirm its bilirubin reductase activity through fluorescence assays and metabolomics. When analysing human gut metagenomes, we found that bilirubin reductase was nearly universally present in healthy adults, while the prevalence of the gene was much lower in patients with inflammatory bowel disease (IBD) and in infants, especially during the first few months of life when infants are most susceptible to developing jaundice. This study fills a long-standing knowledge gap in the haem degradation pathway and provides a building block to understand disorders of serum bilirubin homeostasis such as jaundice.

## Results

### Identification of a putative bilirubin reductase

**Extended Data Fig. 1:**
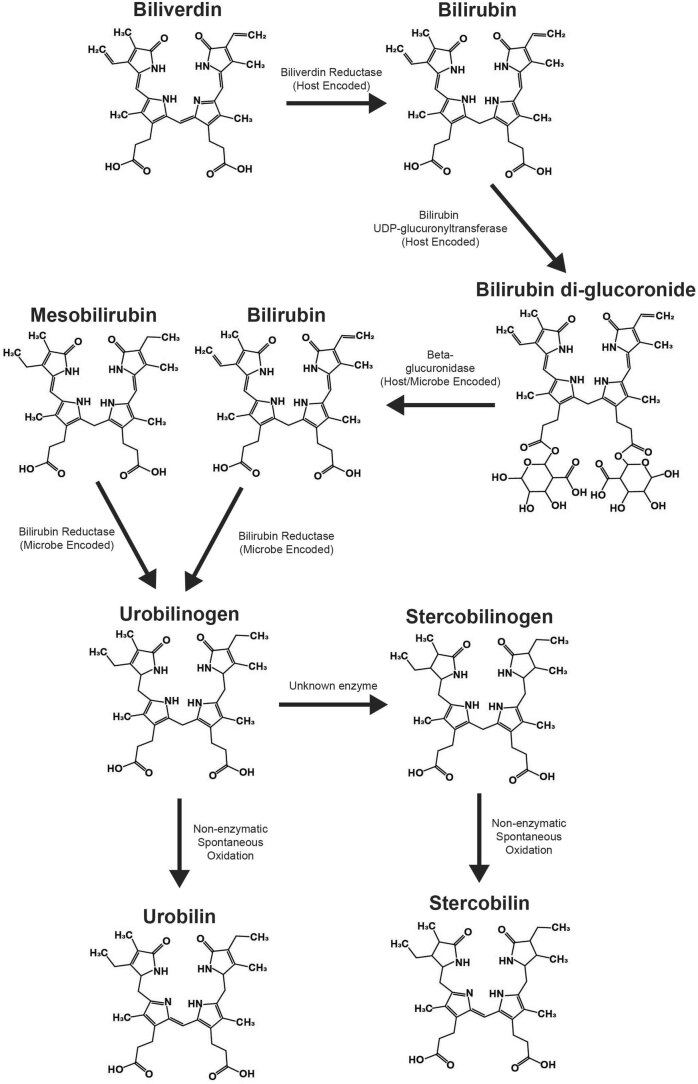
Diagram of the heme degradation pathway in humans. Chemical structures are shown for each intermediate metabolite along with the names of the enzymes involved and if they are host or bacterially encoded.

Our goal was to pinpoint the gene encoding bilirubin reductase through experimental screening and comparative genomics. The bilirubin reductase enzyme is probably an oxidoreductase that acts on carbon–carbon double bonds (Enzyme Commission (EC): 1.3.-.-; Fig. 1b and Extended Data Fig. 1). In addition, this gene should be present in the genomes of species that can reduce bilirubin and absent in those that cannot. By examining closely related species with different abilities to reduce bilirubin, we aimed to identify a specific gene responsible for this activity.

**Extended Data Fig. 2:**
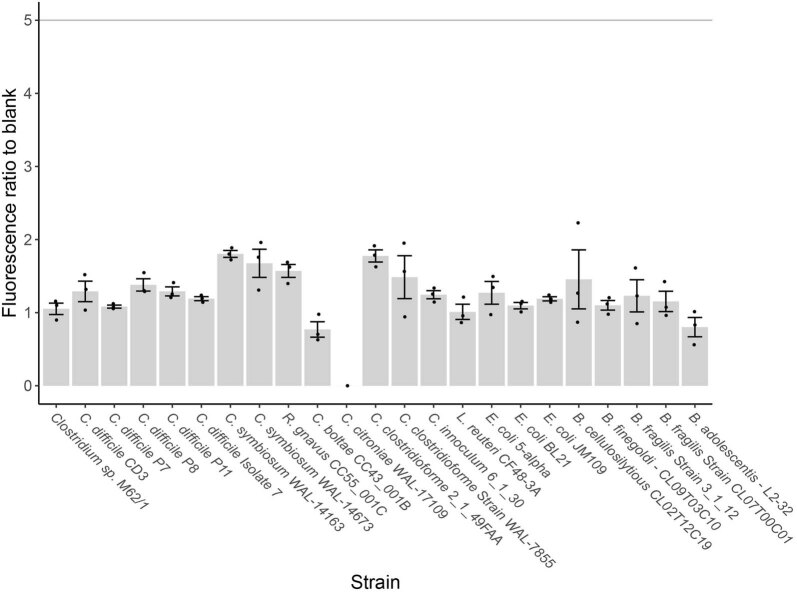
Negative controls for reduction screening. Plots show corresponding fluorescence assays for experiments shown in Fig. 1C, without bilirubin added to the culture. Individual data points from n = 3 independent biological replicates are shown as black points and the bar heights indicate the mean values of the replicates. Error bars indicate one standard error above and below the mean values. The gray line marks a ratio of 5 for the fluorescence to the abiotic media sample. Data was not obtained for C. citroniae WAL-17109.

To identify gut microbial species that were putatively capable of bilirubin reduction, we used a fluorescence assay on extracts from bacteria grown in media with bilirubin. The premise of the assay is that the unstable potential products of bilirubin reduction, urobilinogen and stercobilinogen, can be readily oxidized to the more stable urobilin and stercobilin with the addition of iodine (Extended Data Fig. 1), and measured via fluorescence, while non-protein-bound bilirubin is not fluorescent^18^. As a positive control, we showed that the known bilirubin reducer, *Clostridioides difficile* CD3, returned a positive result in the fluorescence assay and that no fluorescence was produced when no bilirubin was added to the culture (Fig. 1c and Extended Data Fig. 2)^19^.

To search for other potential bilirubin-reducing species, we grew various bacteria from the major phyla of the human gut microbiome, prioritizing species previously reported to reduce bilirubin and their taxonomic relatives, in media supplemented with bilirubin and assayed for urobilin with fluorescence. Using our fluorescence assay, we identified putative bilirubin reduction activity in nine strains, including three species that were previously not known to be capable of bilirubin reduction: *Clostridium symbiosum* (strains WAL-14163 and WAL-14673), *Clostridium* sp. M62/1 and *Ruminococcus gnavus* CC55_001C (Fig. 1c and Extended Data Fig. 2). In addition, 13 gut species did not return a positive fluorescence result (Fig. 1c). Of the tested species, all bilirubin reducers were found to be in the class Clostridia of the phylum Firmicutes. However, close relatives of these reducers, such as *Clostridium clostridioforme* 2_1_49FAA and *Clostridium bolteae* CC43_001B, were not found to be reducers.

We leveraged the variability in bilirubin reduction between closely related strains to identify candidate bilirubin reductase genes through a comparative genomics analysis. Our analysis focused on the genomes of five putative bilirubin-reducing strains and five closely related strains that did not show any bilirubin reduction activity in our fluorescence assay. Among the ten genomes analysed, a total of 6,256 orthogroups were identified, of which 389 were predicted to be putative oxidoreductases. Only two of these orthogroups fit the same pattern of presence and absence in the strains as the bilirubin reduction phenotype. One of them was predicted to be a 4-hydroxy-3-methylbut-2-enyl diphosphate reductase (EC: 1.17.1.2), which is an enzyme that acts on CH or CH_2_ groups and is involved in isoprenoid biosynthesis but did not meet the proposed requirements for a bilirubin reductase enzyme^20^. The other reductase orthogroup was an unannotated enzyme found to be homologous to 2,4-dienoyl-CoA reductase (EC: 1.3.1.34), an oxidoreductase that reduces carbon–carbon double bonds, similar to the expected bilirubin reduction reaction.

**Fig. 2:**
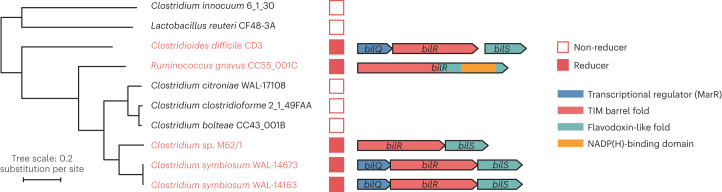
Putative bilirubin reductase operons. The phylogenetic tree shows the relationship between five bilirubin reducers and five non-reducers. Genes are represented as arrows. Genes are coloured to show predicted domains.

**Extended Data Fig. 3:**
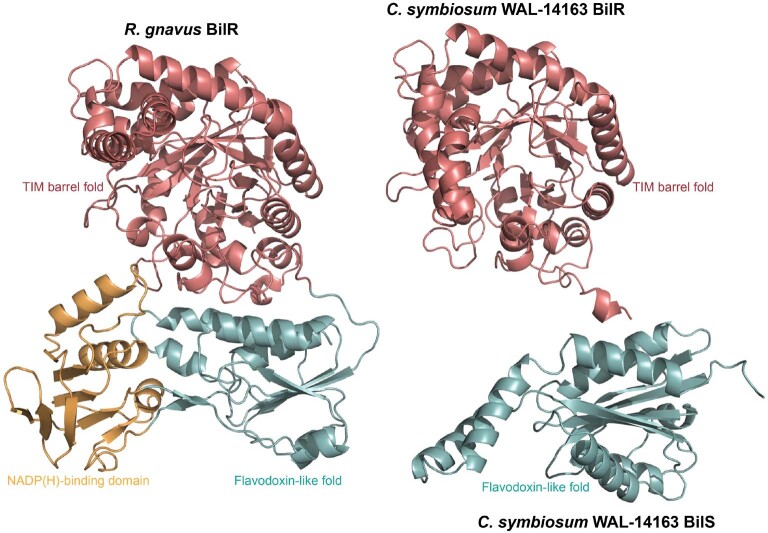
AlphaFold predicted BilR and BilS structures. The *C. symbiosum* BilR and BilS were aligned to the *R. gnavus* BilR structure and are shown in the same orientation to show the similar fold structure. The structures are colored based on the predicted protein domains.

**Extended Data Fig. 4:**
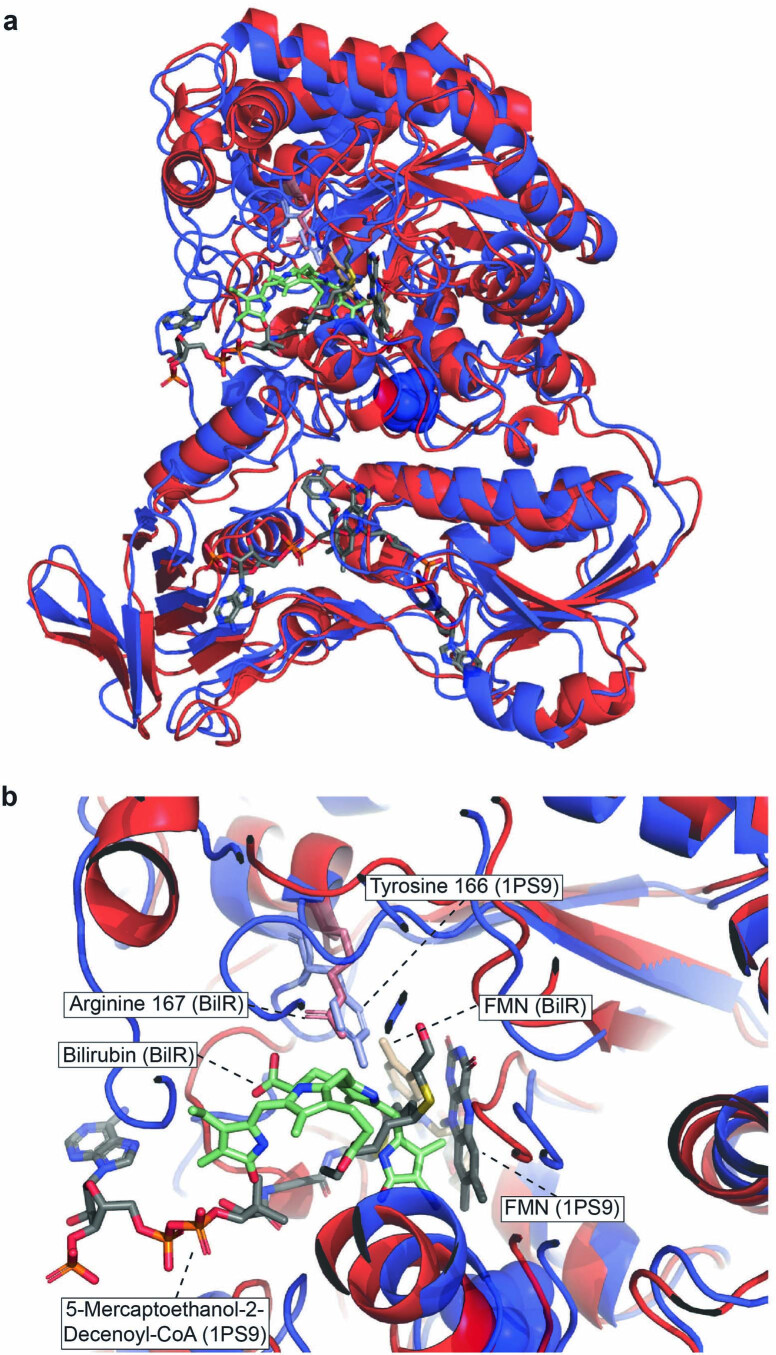
Structural alignment of the predicted BilR and 1PS9 structures. **a)** Alignment of the full structures of 2,4-Dienoyl CoA reductase (1PS9) structure (blue) and the AlphaFold predicted structure of the *R. gnavus* BilR protein (red). **b)** Visualization of the active site on the aligned proteins using the same color scheme. The Arginine 167 residues from the BilR is highlighted in light red and the Tyrosine 166 residues from the 1PS9 protein is highlighted in light blue. The metabolites and residues of interest are labeled.

We then analysed the operons that contained the putative bilirubin reductases. Three versions of a putative bilirubin reductase operon were identified, consisting of different combinations of three genes hereafter referred to as *bilQ*, *bilR* and *bilS* (Fig. 2). *bilR* is the putative bilirubin reductase, *bilS* is a flavodoxin-like protein and *bilQ* is a MarR family transcriptional regulator. *Clostridioides difficile* CD3 and both strains of *Clostridium symbiosum* contained versions of the operon that included all three genes. *Clostridium* sp. M62/1 had only *bilR* and *bilS* and *Ruminococcus gnavus* CC55_001C had just *bilR*. The *Ruminococcus gnavus* CC55_001C *bilR* differs in that it encodes two extra C-terminal domains compared with the other four *bilR* genes (Extended Data Fig. 3). The N-terminal domain of the *Ruminococcus gnavus* CC55_001C BilR protein is predicted to be a triose-phosphate isomerase (TIM) barrel fold, and it is homologous to the full protein sequences of other *bilR* genes. The two extra domains of the *Ruminococcus gnavus* CC55_001C BilR are a flavodoxin-like domain and an NADP(H)-binding domain. There was no sequence homology detected between the flavodoxin-like domain encoded by the *Ruminococcus gnavus* CC55_001C *bilR* and the flavodoxin-like protein encoded by *bilS*, although they are predicted to have the same fold and are probably involved in electron transfer. The BilR of *Ruminococcus gnavus* CC55_001C was predicted to belong to the Old Yellow Enzyme family (COG1902) and exhibits clear homology to *Escherichia coli* 2,4-dienoyl-CoA reductase (Protein Data Bank (PDB): 1PS9, protein identity = 29.82%, Root Mean Square Deviation of predicted BilR and 2,4-dienoyl-CoA reductase = 2.79, normalized template modelling score 0.90; Extended Data Fig. 4), an enzyme that catalyses the reduction of both 2-*trans*,4-*cis* and 2-*trans*,4-*trans*-dienoyl-CoA thioesters^21^. This homology supports the identification of BilR as a putative bilirubin reductase, as bilirubin reductase is hypothesized to be an oxidoreductase that can act on the multiple CH–CH groups of bilirubin.

### BilR confers bilirubin reductase activity

**Fig. 3:**
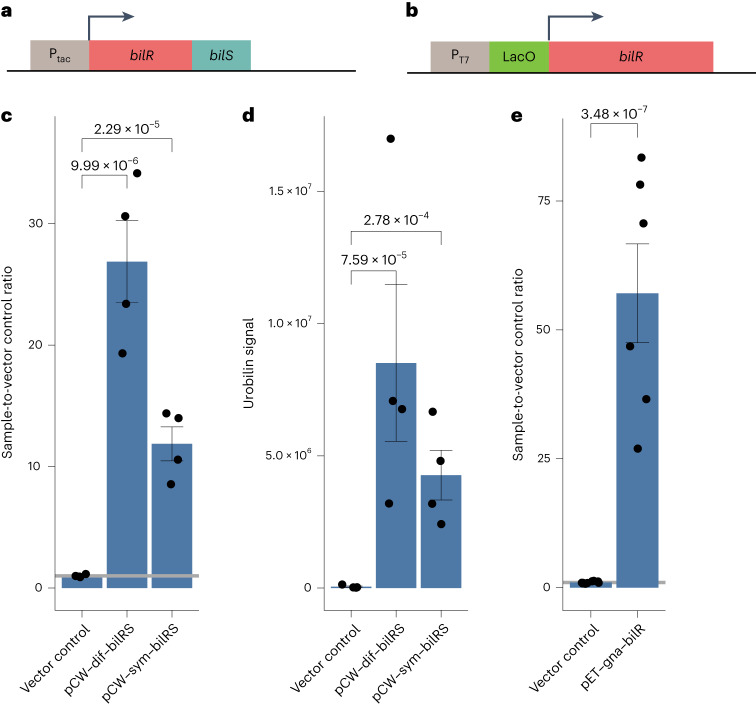
Confirmation of *bilR(S)* bilirubin reductase activity. **a**, Schematic of the *Clostridium symbiosum* and *Clostridioides difficile bilRS* construct. P_tac_ is a tac-promoter, P_T7_ is a T7 promoter, and LacO is a lac operator. **b**, Schematic of the *Ruminococcus gnavus bilR* construct. **c**, Fluorescence assay comparing bilirubin reduction activities of *E. coli* 10-beta transformed with the empty vector, *Clostridium symbiosum* construct and *Clostridioides difficile* construct (two-sample one-sided *t*-test). **d**, Metabolomics confirmation of urobilin production (two-sample one-sided *t*-test). **e**, Fluorescence confirmation of the bilirubin reduction activity of *E. coli* 10-beta transformed with the *Ruminococcus gnavus bilR* construct (two-sample *t*-test). The bar heights show the mean of *n* = 4 independent biological replicates used to generate the plots and statistics in **c** and **d**. A total of *n* = 3 independent biological replicates were used to generate the plots and statistics in **e**. Error bars indicate 1 s.e. above and below the mean. *P* values of a two-sample one-sided *t*-test to determine whether the mean of the samples is greater than the mean of the vector controls are provided above the brackets. Individual data points are indicated by black points on each plot.

**Extended Data Fig. 5:**
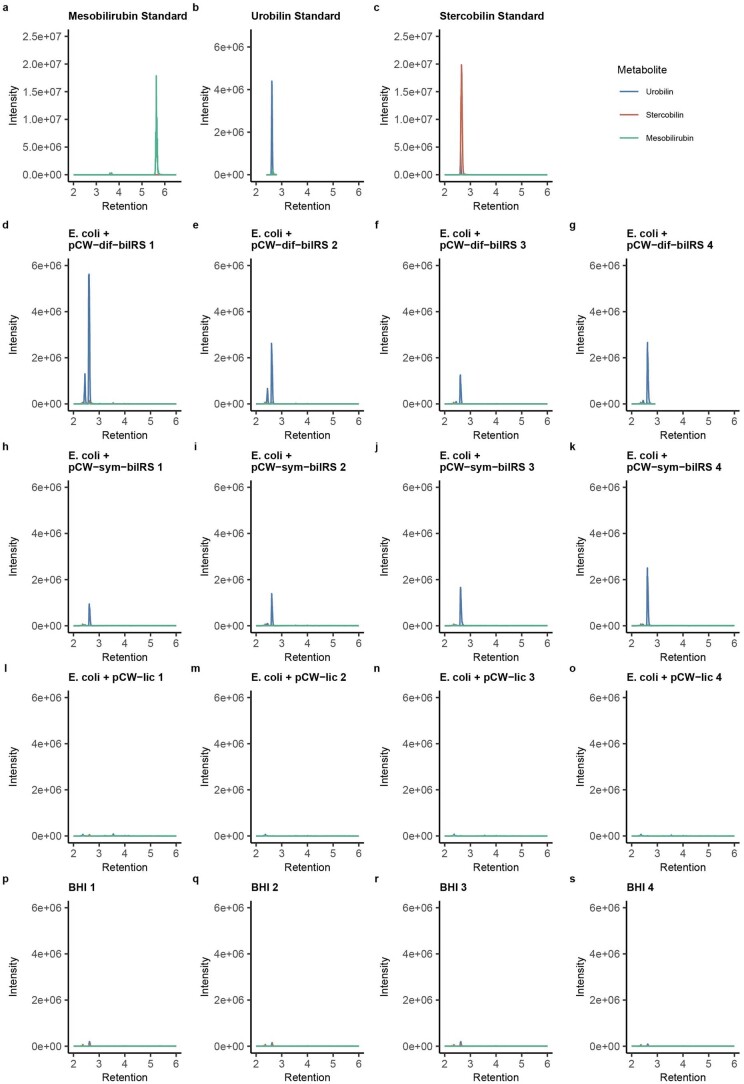
Metabolomics confirmation of bilirubin reduction. Extraction ion chromatograms for the detection of Mesobilirubin (m/z 589.3 →m/z 301.2) Urobilin (m/z 591.4→ m/z 343.2) and Stercobilin (m/z 595.4→m/z 345.2). **a)** Mesobilirubin standard. **b)** Urobilin standard. **c)** Stercobilin standard. **d-g)** pCW-dif-bilRS biological replicates. **h-k)** pCW-sym-bilRS biological replicates. **l-o)** Vector control samples. **p-s)** BHI media control samples. Panels a and a have different Y and X axis scales compared to the other plots to show the corresponding peaks.

**Extended Data Fig. 6:**
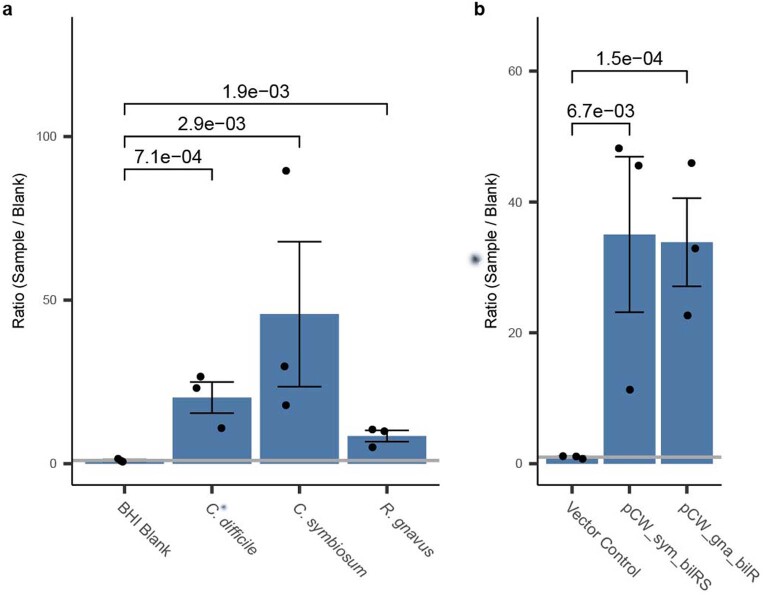
Confirmation of reduction of bilirubin, mesobilirubin to urobilin. **a)** Fluorescence assay comparing mesobilirubin reduction activity of C. difficile CD3, C. symbiosum WAL-14163, and R. gnavus to an abiotic media controls with mesobilirubin added. **b)** Fluorescence assay comparing mesobilirubin reduction activity of heterologously expressed C. symbiosum bilRS in E. coli to the vector control (two-sample one-sided t-test). Bar heights in plots indicate the mean values of n = 3 independent biological replicates. The gray line marks a ratio of 1, equivalent to the mean control fluorescence. Individual data points are indicated by black points on each plot. Error bars indicate one standard error above and below the mean values. P-values of a two-sample one-sided t-test testing if the mean of the samples are greater than the means of the controls are shown on panels a and b.

**Extended Data Fig. 7:**
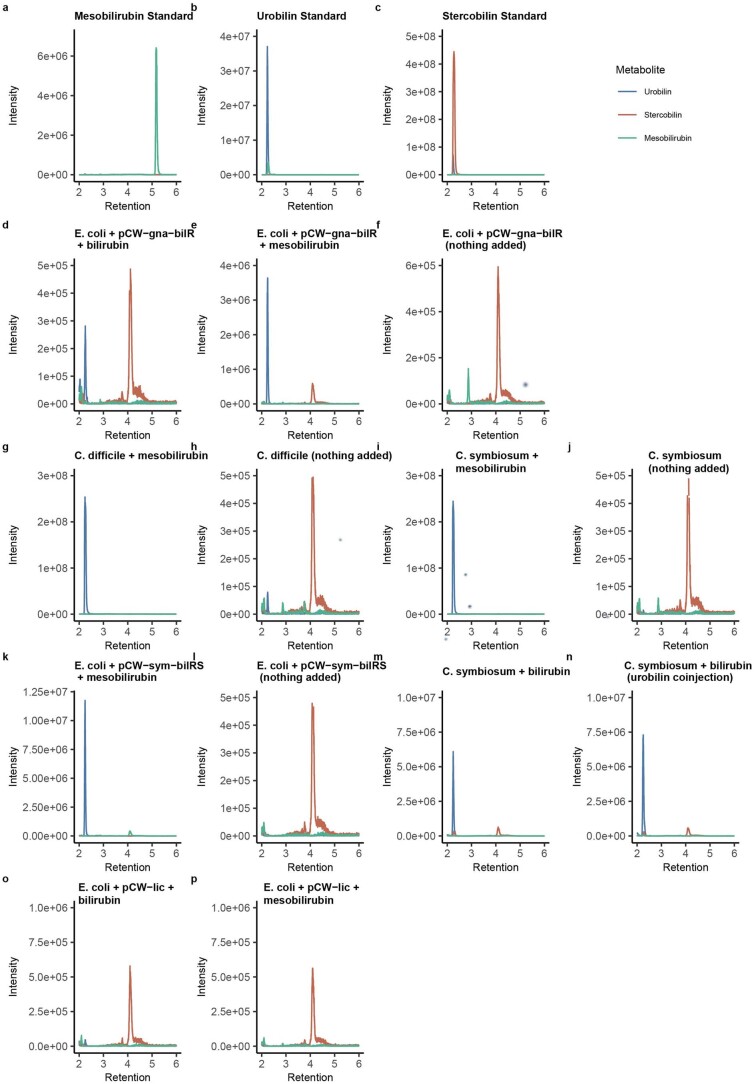
Metabolomics confirmation of mesobilirubin reduction. Extraction ion chromatograms for the detection of Mesobilirubin (m/z 589.3 →m/z 301.2) Urobilin (m/z 591.4→ m/z 343.2) and Stercobilin (m/z 595.4→m/z 345.2). **a)** Mesobilirubin standard. **b)** Urobilin standard. **c)** Stercobilin standard. **d-f)** E. coli transformed with pCW_gna_bilRS incubated with bilirubin (d), mesobilirubin (e) or nothing (f). **g-h)** C. difficile incubated with mesobilirubin (g) or nothing (h). **i-j)** C. symbiosum incubated with mesobilirubin (i) or nothing (J). **k-l)** E. coli transformed with pCW_sym_bilRS incubated with mesobilirubin (k) or nothing (l). **m-n)** C. symbiosum incubated with bilirubin (m) and incubated with bilirubin and co-injected with the urobilin standard (n). **o-p)** E. coli strains transformed with empty pCW vector incubated with bilirubin (o) and mesobilirubin (p).

Once BilR was identified as a candidate bilirubin reductase, we sought to experimentally validate that it is sufficient to confer bilirubin reductase activity through heterologous expression in *E. coli*. The *bilRS* genes from *Clostridium symbiosum* and *Clostridioides difficile* (short *bilRS*) and *bilR* from *Ruminococcus gnavus* (long *bilR*) were separately cloned into a pCW–lic vector and then transformed into *E. coli* 10-beta or into a pET-28a(+) vector and transformed into *E. coli* T7 express lysY/Iq (Fig. 3a,b). To validate that the fluorescence assay could be used as a proxy for bilirubin reduction to urobilinogen, we assayed for the reduction of bilirubin to urobilinogen via fluorescence assay and verified the results with liquid chromatography and tandem mass spectrometry (LC–MS/MS; Fig. 3c,d). The strains transformed with the *Clostridioides difficile* and *Clostridium symbiosum* BilRS both showed significant fluorescence after being incubated with bilirubin, and the presence of urobilin was observed in the corresponding LC–MS samples (fluorescence assay: *Clostridioides difficile* BilRS *P* = 9.99 × 10^−6^, *Clostridium symbiosum* BilRS *P* = 2.29 × 10^−5^, two-sample *t*-test on log_2_-transformed data; metabolomics: *Clostridioides difficile* *P* = 7.59 × 10^−5^, *Clostridium symbiosum* *P* = 2.78 × 10^−4^, two-sample *t*-test on log_2_-transformed data (urobilin *m/z* 591.4→*m/z* 343.2)). The samples did not have any peaks indicating that stercobilin was present (Extended Data Fig. 5), suggesting that the assay could be used as a reliable indication of bilirubin reduction to urobilinogen. The long BilR construct was also capable of reducing bilirubin to urobilinogen (fluorescence assay: *Ruminococcus gnavus* BilR *P* = 3.48 × 10^−7^, two-sample *t*-test on log_2_-transformed data; Fig. 3e), and *E. coli* with the empty vector backbone pCW–lic did not reduce bilirubin (*P* = 0.167, one-sided *t*-test on log_2_-transformed data against the abiotic media control). Neither mesobilirubin (*m/z* 589.3→*m/z* 301.2) nor stercobilin (*m/z* 595.4→*m/z* 345.2) was consistently detected (Extended Data Fig. 5). Native bilirubin reducers and *E. coli* expressing *bilRS* were also able to reduce mesobilirubin to urobilinogen suggesting that the enzyme can act on all four double bonds in bilirubin (Extended Data Figs. 6a,b and 7). These results show that BilR is sufficient to reduce bilirubin or mesobilirubin to urobilinogen (Extended Data Fig. 1).

### Delineation of the BilR clade

**Fig. 4:**
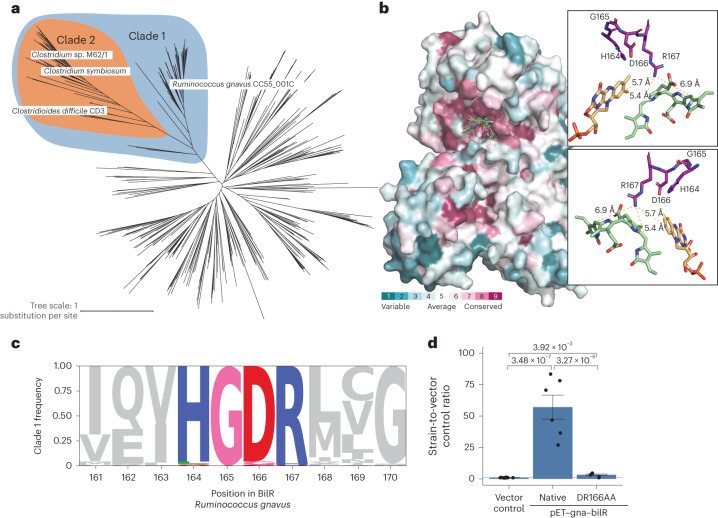
Identification of a *bilR* clade. **a**, Gene tree constructed from putative *bilR* sequences and related reductases from the Old Yellow Enzyme family. Experimentally confirmed bilirubin reducers are labelled in the tree. Clade 1 indicates the overall *bilR* clade, and clade 2 indicates the short *bilR* clade. The same tree with all bootstrap values and species labels is included in Extended Data Fig. 8. **b**, AlphaFold-predicted structure of the *Ruminococcus gnavus* BilR with sequences coloured to show their degree of conservation within clade 1 based on a ConSurf conservation analysis. A docked bilirubin (green) and FMN (orange) molecule are shown on the structure, and the insets show the potential site of interaction between the R167 residue and the putative enzyme substrates. The HGDR residues are coloured purple in the insets. **c**, Diagram showing the conservation of positions within the clade 1 BilR sequences. The conserved HGDR motif positions are coloured. **d**, Fluorescence assay results comparing the bilirubin reductase activities in *E. coli* 10-beta transformed with the vector control, *Ruminococcus gnavus bilR* and *Ruminococcus gnavus bilR* with the Aspartic Acid, Arginine (DR) residues at position 166–167 mutated to Alanine, Alanine (AA). The bar heights are based on the mean of *n* = 6 independent biological replicates used to generate the plots and statistics in **d**. Error bars indicate 1 s.e. above and below the mean values. *P* values of a two-sample one-sided *t*-test to determine whether the mean of the native *Ruminococcus gnavus bilR* was higher than the mean of the vector control or mutant are provided. Individual data points are indicated by black points on each plot.

**Extended Data Fig. 8:**
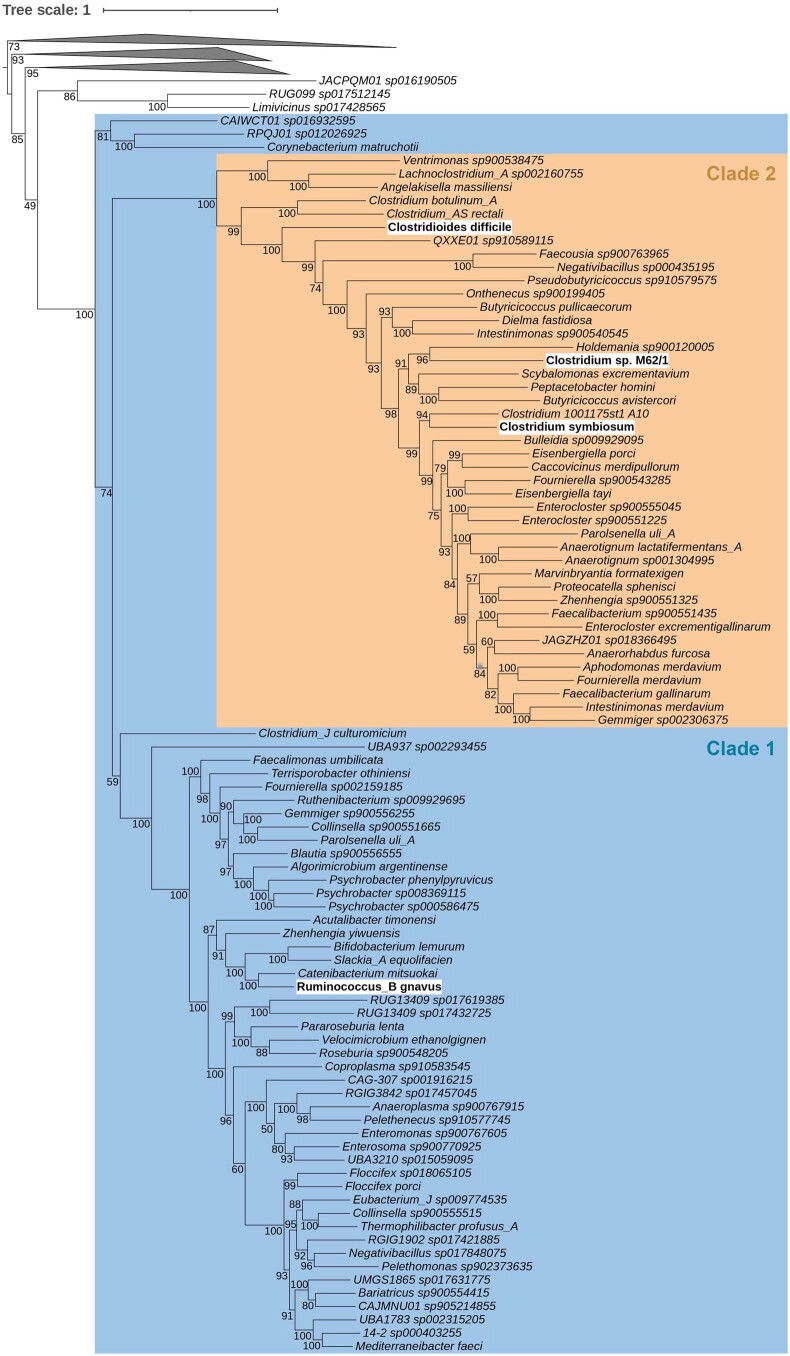
Gene tree of bilR clade. This tree is the same as the tree represented in Fig. 4a. Bootstrap values are included based on 1000 bootstrap iterations. Species with experimentally confirmed BilR genes are shown with white backgrounds. Clade 1 of the bilR genes is highlighted in blue, and Clade 2 is highlighted in orange. Clades not containing bilR genes are collapsed.

To delineate the BilR sequences from other members of the Old Yellow Enzyme family, a combination of structural prediction and sequence conservation was used to identify key residues in the putative BilR sequences and differentiate BilR from the other sequences (Fig. 4a and Extended Data Fig. 8). Our analysis has revealed that one particular clade, referred to as clade 1, is likely to be the bilirubin reductase clade (Fig. 4a and Extended Data Fig. 8). The predicted structure of BilR was compared with the structurally homologous *E. coli* 2,4-dienoyl-CoA reductase enzyme, providing an indication of the putative active site in the BilR enzyme (Extended Data Fig. 4). The Y166 residue in the *E. coli* 2,4-dienoyl-CoA reductase is thought to protonate one of the carbon atoms that form the carbon double bond in the enzyme’s substrate completing the reduction of that bond^21,22^. The similar positioning of the positively charged arginine residue in the predicted BilR structure suggests that this R167 residue or the neighbouring D166 residue may also be acting as a proton donor, completing the reduction reaction after a hydride transfer to the carbon–carbon double bond from a flavin cofactor of NAD(P)H. The D166 and R167 residues, along with the neighbouring H164 and G165 residues, were found to be conserved among the BilR genes from the five confirmed bilirubin-reducing species. A search for this Histidine, Glycine, Aspartic acid, Arginine (HGDR) motif within the Old Yellow Enzyme family revealed that it was nearly universally conserved within a larger clade of proteins that includes all five bilirubin reductases within it (Fig. 4b,c). The conservation of these residues within the clade 1 sequences (Fig. 4a), combined with the similar positioning of these residues to the active site residues in the *E. coli* 2,4-dienoyl-CoA reductase enzyme, suggests that clade 1 contains the bilirubin reductase enzymes.

**Extended Data Fig. 9:**
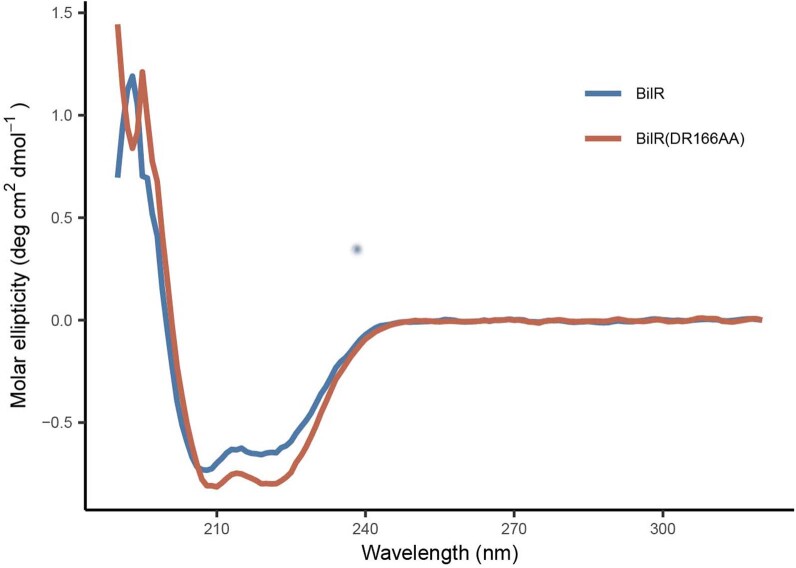
Circular dichromism confirmation of mutant bilR structural similarity. Circular dichroism spectra for the wild type BilR protein (blue) and DR166AA mutant BilR protein (red).

To confirm that clade 1 is composed of bilirubin reductase enzymes, we performed experiments to investigate the functional impact of the D166 and R167 residues on the enzyme activity. This included mutating the residues in question to D166A and R167A and heterologously expressing the mutated protein to measure the impact on the enzyme activity. The mutated protein showed similar structural properties as the wild-type protein, confirming that the mutation did not lead to a major disruption in the overall folding of the enzyme (Extended Data Fig. 9). When expressed in *E. coli*, the mutated protein showed a significantly lower bilirubin reductase activity than the wild-type protein (*P* = 3.27 × 10^−6^, two-sample *t*-test; Fig. 4d). This experimental approach provides direct evidence that the D166 and R167 residues in the active site are critical for bilirubin reduction, a characteristic that is specific to bilirubin reductase enzymes and distinct from that of other members of the Old Yellow Enzyme family, supporting the assignment of clade 1 as bilirubin reductase (Fig. 4a).

**Fig. 5:**
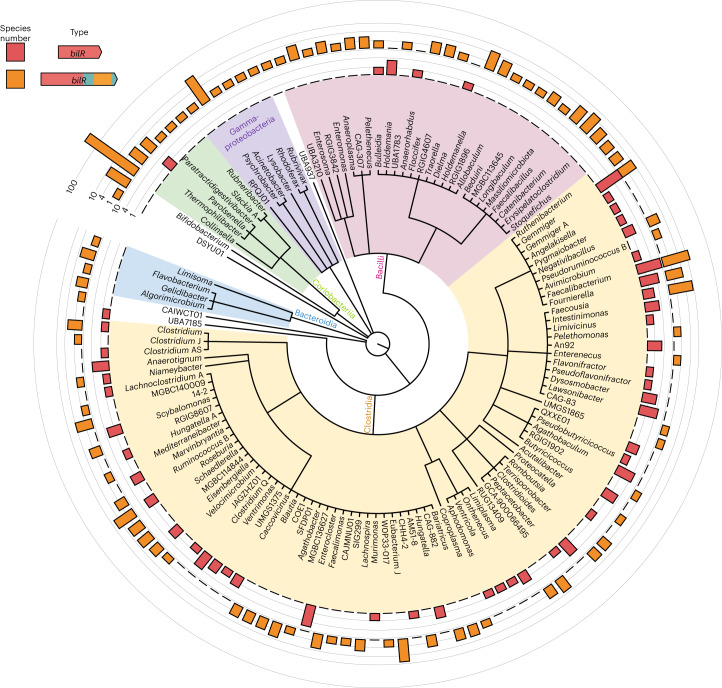
Taxonomic distribution of bilirubin reductase. The cladogram shows the relationships between different taxa with detected bilirubin reductase genes. The outer rings show the presence of the short *bilR* gene (red) and long *bilR* gene (orange).

We then examined the taxonomic distribution and diversity of bilirubin reducers, identifying putative bilirubin reductases in 658 of the representative genomes in the Genome Taxonomy Database (GTDB; Fig. 5 and Supplementary Table 1). The distribution of *bilR* genes is largely in agreement with previously reported bilirubin-reducing species, with species such as *Clostridium ramosum* and *Clostridioides difficile* having predicted *bilR* genes. While the representative genome of *Clostridium perfringens* does not have bilirubin reductase, 137 out of 315 genomes assigned to the *Clostridium perfringens* species have it, suggesting that bilirubin reduction is a strain-specific feature. Notably, no species within the Bacteroidaceae family were predicted to be bilirubin reducers, conflicting with a previous report from 1972, but this could be due to this being a strain-specific feature or incorrect taxonomic assignment^17^.

The long variant of the *bilR* gene is widely distributed across multiple phyla, while the short variant seems to be more exclusive to Firmicutes, being seen only in the *Parolsenella* genus outside of Firmicutes. The majority of *bilR* genes were identified in Firmicutes species, including common human gut species such as *Roseburia intestinalis*, *Roseburia inulinivorans* and *Faecalibacterium prausnitzii*, with a mixture of both *bilR* versions being seen. The short variant of the *bilR* gene is monophyletic (clade 2 in Fig. 4a), suggesting that there has been extensive horizontal gene transfer during the evolution of *bilR*, particularly in Firmicutes. Some *bilR* genes were found in bacteria from the Flavobacteriales, which are typically found in aquatic and soil environments, suggesting a possible role for bilirubin reductase in breaking down bilirubin or similar metabolites in other environments. Nine species in the genus *Bifidobacterium* were predicted to be bilirubin reducers and were isolated from the faeces of non-human animals such as chickens, lemurs and monkeys. This implies that the bilirubin-reducing capacity of the human gut microbiota is largely influenced by the abundance of Firmicutes-associated bilirubin reducers.

### Bilirubin reductase is often absent in the neonate gut

**Fig. 6:**
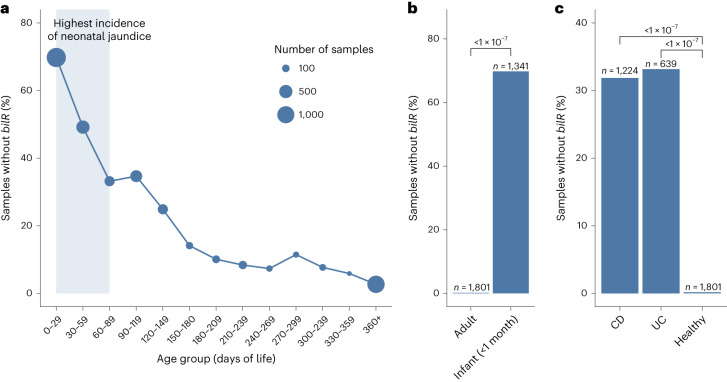
Presence of *bilR* in the human gut during development and disease. **a**, Percentage of infant gut metagenomes missing *bilR* during their first year of life. The period of highest jaundice susceptibility is indicated by a shaded blue area on the plot. **b**, Comparison of *bilR* absence in samples from healthy adults and infants in their first month of life. **c**, Comparison of the percentage of samples with no *bilR* detected from healthy adults and adults with IBD (Crohn’s disease (CD) or ulcerative colitis (UC)). The number of metagenomic samples included in each dataset is indicated above each bar. The *P* values for each comparison show the results of a test of equal proportions to determine whether the fraction of samples with no *bilR* detected was different between groups, without adjusting for multiple testing.

**Extended Data Fig. 10:**
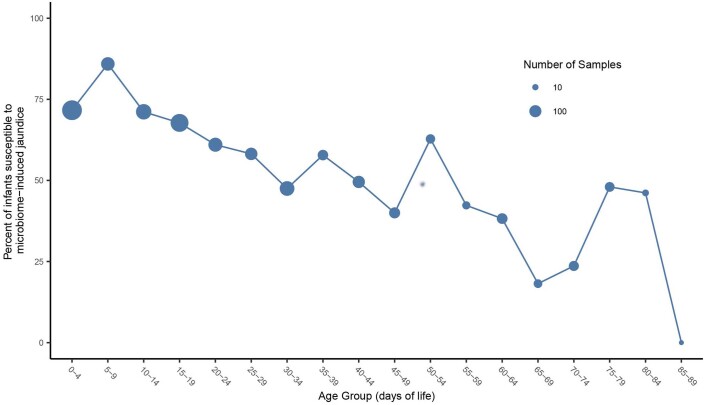
Frequent absence of bilR during the first three months of life. Plot showing the percent of samples within each five-day age bin with no bilirubin reductase detected. Points are sized based on the number of samples included in each bin.

To examine the relationship of bilirubin reductase to age and health, we performed a large-scale analysis of human gut metagenomes. We examined the presence and absence of bilirubin reductase across 4,296 infant gut metagenomes from the first year of life (Supplementary Table 2). We saw a stark trend in which bilirubin reductase was often absent in infants during the first few months (Extended Data Fig. 10), when neonatal jaundice risk is highest, but was mostly present by the end of the first year of life only^23^ (Fig. 6a). While various factors are likely to influence the serum bilirubin levels in infants, the correspondence between the period of life in which we observe the most samples missing bilirubin reductase and the risk of neonatal jaundice suggests a strong connection between the microbiome composition and the development of jaundice in infants.

### Bilirubin reduction is a core function of the human gut

Serum bilirubin levels are significantly altered in patients with IBD, suggesting that the bilirubin-metabolizing microbiome is disrupted in this disease^24^. When examining metagenomes from healthy adults, we found that bilirubin reductase was absent in only 0.1% of the 1,801 samples analysed, a significantly lower fraction of samples (*P* < 2.2 × 10^−16^, test of equal proportions) than was seen in the young infants (Fig. 6b). Compared with healthy adults, patients with IBD have been observed to have lower levels of urobilin and have been observed to form pigmented gallstones containing high levels of bilirubin^25,26^. Bilirubin reductase was absent in a significantly higher fraction of samples from patients with Crohn’s disease (*P* < 2.2 × 10^−16^, test of equal proportions) or ulcerative colitis (*P* < 2.2 × 10^−16^, test of equal proportions) compared with healthy samples (Fig. 6c). This provides evidence that the microbial taxonomic alterations associated with IBD and other inflammatory diseases may be having a direct impact on the bilirubin reduction activity of the gut microbiome.

## Discussion

Despite the identification of urobilin as the yellow pigment in urine more than 125 years ago, the enzyme responsible for its production has remained a mystery^27^. Though it was previously thought that multiple enzymes were involved in the reduction of bilirubin, our results support the finding that a single enzyme performs the reduction of bilirubin to urobilinogen^19^. The identification of bilirubin reductase allowed us to profile its abundance in 7,960 metagenomes, showing that bilirubin reduction is a core feature of a healthy adult human microbiome and that neonates are often missing bilirubin reductase during the period of the highest incidence of neonatal jaundice^28,29^. Furthermore, we found that the prevalence of bilirubin reductase is decreased in patients with IBD.

Our experiments have shown that bilirubin reductase can perform the complete reduction of bilirubin to urobilinogen and has the potential to act on multiple substrates, placing this single enzyme in a central role in bilirubin homeostasis. The importance of this process is further highlighted by the limited capacity of the UGT1A1 enzyme in the liver to conjugate serum bilirubin, a rate-limiting step in haem degradation^30^. In addition, conjugated bilirubin is thought to be readily deconjugated by gut microorganisms, and the activity of microorganisms on conjugated bilirubin has been shown to be limited, suggesting that bilirubin reduction is the key step that determines the degree to which haem degradation by-products are reabsorbed or excreted^5,11,19,31^.

While the discovery of bilirubin reductase illuminates a key gap in our understanding of the health-relevant steps of haem excretion, there are additional aspects of the pathway that require elucidation. First, while it is likely that bilirubin is deconjugated by beta-glucuronidases before it is reduced by BilR, additional experiments with bilirubin diglucuronide are necessary to confirm the order of the pathway^19^. Second, there is an additional step in the pathway downstream of bilirubin reduction performed by the gut microbiome where the enzyme is still unknown: the reduction of urobilinogen to stercobilinogen. Similar to bilirubin, urobilinogen may serve as a terminal electron acceptor for microorganisms encoding these reductases, providing them a competitive advantage in the anaerobic environment of the gut.

With the knowledge of the species, genes and enzymes involved in bilirubin reduction, future research can now focus on the extent to which gut microbial bilirubin metabolism affects serum bilirubin homeostasis and the role of bilirubin reduction in health and disease. Our finding that bilirubin reductase is often absent in young infants supports the hypothesis that neonatal jaundice is exacerbated by the absence or low abundance of bilirubin-reducing microorganisms in the gut^11,12^. Multiple factors related to both the host and microbiome undoubtedly contribute to the development of jaundice. To address these factors, new cohort studies that simultaneously measure serum bilirubin, faecal urobilinoids and the absolute abundance of bilirubin-reducing bacteria in infants will be needed to disentangle their roles in this disease^32,33^. In addition to neonatal jaundice, bilirubin reduction by gut microorganisms is important to overall health because serum bilirubin concentrations have been observed to be correlated inversely with adiposity and cardiovascular disease^10,34^. Urobilin concentrations also have an impact on human health, being positively correlated with insulin resistance and heart failure^9,10^. This further highlights the complexity and importance of the haem degradation pathway, confirming the key role that bilirubin reductase plays in determining the balance of multiple health-relevant metabolites.

The differences observed between the metagenomes of healthy individuals and those of patients with IBD highlight the potential disruption of bilirubin metabolism in IBD. The lowered prevalence of bilirubin-reducing bacteria in patients with IBD leads us to hypothesize that the disruption of bilirubin metabolism combined with the previously established increase in unconjugated primary bile acids in patients with IBD could contribute to the increased incidence of calcium bilirubinate gallstones that has been observed in these patients^25,35^. While these results suggest a role for bilirubin-metabolizing microorganisms in these diseases, more work is needed before any conclusions can be made. Our work fills a long-standing knowledge gap, providing foundational knowledge that will serve as the basis of future investigations of the importance of bilirubin metabolism in human health.

## Methods

### Culture methods

#### Anaerobic bacteria

Bacterial strains were obtained from the NIH Biodefense and Emerging Infections Research Resources Repository (BEI). Strains were inoculated from a glycerol stock and grown under anaerobic conditions (90% N_2_, 5% CO_2_, 5% H_2_) at 37 °C in an anaerobic chamber (Coy Laboratory Products). Strains were grown in 200 ml of liquid brain–heart infusion (BHI) broth (Research Products International, B11000) or yeast casitone fatty acid broth with 4.4 mg per 100 ml sterile filtered bilirubin dissolved in dimethylsulfoxide (DMSO, Sigma-Aldrich). The bacteria were grown anaerobically at 37 °C for 24 h before the chloroform extraction was performed.

#### Transformed *E. coli*

Transformed *E. coli* strains were inoculated from an agar colony plate into 300 ml BHI media with 100 μg ml^−1^ carbenicillin (GoldBio, 00901C103) or 50 μg ml^−1^ kanamycin and 100 μM isopropyl β-d-1-thiogalactopyranoside (IPTG, GoldBio, 221105I2481) and shaken aerobically at 37 °C for 16 h. The bacteria were then pelleted by centrifugation at 3,260 × *g* for 6 min and moved to the anaerobic chamber. The pellet was resuspended in 200 ml of BHI with 4.4 mg per 100 ml sterile filtered bilirubin dissolved in DMSO, 100 μg ml^−1^ carbenicillin or 50 μg ml^−1^ kanamycin, and 100 μM IPTG to induce expression of the cloned *bilR* gene(s). The strains were incubated anaerobically at 37 °C for 24 h before chloroform extraction was performed.

### Measuring bilirubin reduction

#### Fluorescence assay

Bacterial cultures were removed from the anaerobic chamber and pelleted by centrifugation at 3,260 × *g* for 6 min. The supernatant was filtered through a non-sterile 0.22 μm filter. Chloroform (5 ml; Sigma-Aldrich) was added to the filtered supernatant and separated via organic extraction in a separatory funnel. The resultant chloroform–urobilinogen solution was air-dried until the chloroform was completely evaporated, producing the extract used in the subsequent fluorescence assays and LC–MS/MS.

Because the urobilinogen product of bilirubin reduction is unstable in oxygen, measuring urobilinogen itself was infeasible. Therefore, to measure bilirubin reduction, urobilinogen was oxidized to urobilin via the addition of iodine. The urobilin was quantified by adding zinc acetate solution to form a fluorescent zinc–urobilin complex^36–38^. However, this assay also detects stercobilin, which fluoresces at the same wavelength as urobilin^39,40^. To differentiate the two potential products, the fluorescence assay was verified by mass spectrometry.

The instability of urobilinogen requires this assay to be performed immediately after the chloroform from the chloroform extraction had evaporated. The extracts were rehydrated using 600 µl of deionized water. A 400 µl aliquot of the rehydrated solution was transferred to a separate tube to continue the assay. To oxidize the urobilinogen to urobilin, 10 µl of 10% povidone–iodine solution (CVS, A47194) was added, followed by 10 µl of a 100 mM cysteine solution to reduce the rest of the iodine and prevent bilirubin oxidation after the addition of Schlesinger’s reagent (545 mM zinc acetate in methanol). Schlesinger’s reagent (400 µl) was added to increase the fluorescence of urobilin. The resulting solution was portioned into 100 µl triplicates or quadruplets onto a 96-well acrylic plate. Each well’s fluorescence was measured with a SpectraMax M5 plate reader using a 495 nm wavelength excitation and 525 nm wavelength emission at medium gain. Samples that produced a fluorescence signal greater than five times the fluorescence of an abiotic media control were identified as reducers. Samples below the five times threshold were considered to be non-reducers.

#### LC–MS/MS

An aliquot of 1 ml of the chloroform solution from the chloroform extraction was taken and allowed to dry completely. The samples were sent with overnight shipping in dry ice to the Duke metabolomics core. To the dried samples was added 60 µl of 10% povidone–iodine solution. The samples were vortexed, then centrifuged at 15,000 × *g* for 5 min at 4 °C. The supernatant was transferred into a 1.7 ml Macherey-Nagel glass vial for the LC–MS/MS injection.

Samples were analysed with the 6500+ QTRAP LC–MS/MS system (Sciex). The Sciex ExionLC ultra-performance liquid chromatography system includes a degasser, an AD autosampler, an AD column oven, a controller and an AD pump. The liquid chromatography separation was performed on an Agilent Eclipse Plus C18 RRHD column (2.1 × 50 mm, 1.8 μm) with mobile phase A (0.2% formic acid in water) and mobile phase B (0.2% formic acid in acetonitrile). The flow rate was 0.35 ml min^−1^. The linear gradient was as follows: 0–0.5 min, 100% A; 4.0–5.5 min, 0% A; and 5.6–7.5 min, 100% A. The autosampler was set at 10 °C, and the column was kept at 35 °C. The injection volume was 5 μl. Mass spectra were acquired under positive electrospray ionization with an ion spray voltage of 5,500 V. The source temperature was 450 °C. The curtain gas, ion source gas 1 and ion source gas 2 were 33, 55 and 60 psi, respectively. Multiple reaction monitoring was used to detect mesobilirubin (*m/z* 589.3→*m/z* 301.2), urobilin (*m/z* 591.4→*m/z* 343.2) and stercobilin (*m/z* 595.4→*m/z* 345.2). A co-injection of *Clostridium symbiosum* incubated with bilirubin and the urobilinogen standard was performed to ensure that the retention times seen in the samples and standards were consistent (Extended Data Fig. 7m,n). All data were analysed in Analyst 1.7.1 software.

### Construct development

#### pCW–sym–*bilRS*

To express the *bilRS* genes from *Clostridium symbiosum* in *E. coli*, the gene was amplified and inserted into the expression vector backbone pCW–lic (Addgene, 26908). A polymerase chain reaction (PCR) amplification using OneTaq Master Mix (New England Biolabs (NEB), M0482) was performed on *Clostridium symbiosum* genomic DNA with forward primer TAAGCACATATGCTGGGAAAGACGAAGGAGGCTC and reverse primer TAAGCAGGTACCGGCAGCGTCCCTGAGT to amplify *Clostridium symbiosum bilRS*. The product was purified with a Monarch PCR and DNA cleanup kit (NEB, T1030) and restricted with enzymes Nde1 (NEB, R0111) and Kpn1-HF (NEB, R3142) using NEBcloner protocols to prepare for ligation into the pCW–lic backbone. The pCW–lic vector backbone was restricted with the same enzymes. pCW–lic was dephosphorylated with antarctic phosphatase (NEB, M0289) to prevent self-ligation. The restricted insert and dephosphorylated backbone were ligated with T4 DNA ligase (NEB, M0202) to form the construct.

#### pCW–dif–*bilRS*

The same protocol was used as for pCW–sym–*bilRS*, with two exceptions. PCR amplification was performed on *Clostridioides difficile* genomic DNA with forward primer TAAGCAGGATCCCAGCTGTGGAGGAAGAATAGGATG and reverse primer TGCTTAAAGCTTCTACTACACAATACTAGCTTTAATCATCATA to amplify *Clostridioides difficile bilRS*. The product was restricted with enzymes BamH1-HF (NEB, R3136) and HindIII-HF (NEB, R3104) before ligating.

#### pCW–gna–*bilR*

The same protocol was used as for pCW–sym–*bilRS*, with two exceptions. PCR amplification was performed on *Ruminococcus gnavus* genomic DNA with forward primer TAAGCAGGATCCGCAGAAAGAAAATGTTAAGGAGAGGCTG and reverse primer TAAGCAGGTACCGAAGGATGTTTCATCCACCTGTACG to amplify *Ruminococcus gnavus bilR*. The product was restricted with enzymes BamH1-HF and Kpn1-HF before ligating.

#### Transformation with pCW constructs

The constructs were individually transformed into NEB 10β competent *E. coli* cells using the manufacturer’s protocol (NEB, C3019). The cells were plated on Luria–Bertani (LB) plates with 100 μg ml^−1^ carbenicillin to select for successfully transformed colonies. The transformed colonies were verified through Sanger sequencing by Azenta.

#### pET28–*bilR*

In an effort to optimize the expression of *bilR*, a second construct was developed using a pET28a(+) vector backbone. To clone the *bilR* gene from *Ruminococcus gnavus* CC55_001C into pET28a(+) for protein expression, the insert was amplified from a glycerol stock of *Ruminococcus gnavus* using a reported protocol^41^. The forward primer used was CGGCAGCCATATGAGATTATTAGAACCAATTAAAG, and the reverse primer was GGCCGCAAGCTTATAAAACGTTTGCTGCC. The vector backbone was amplified from pET28a(+) with the forward primer CGTTTTATAAGCTTGCGGCCGCACTCGAG and the reverse primer ATAATCTCATATGGCTGCCGCGCGGCAC. The resulting insert and vector backbone PCR products were assembled using the NEBuilder HiFi DNA Assembly Master Mix (NEB, E2621S) to yield pET28–BilR. The Q5 High-Fidelity 2× Master Mix (NEB, M0492L) was used in PCR, and NEB Turbo Competent *E. coli* (NEB, C2984H) was used as competent cells in cloning.

#### pET28–*bilR*–166AA

Site-directed mutagenesis was performed using protocols from the Agilent QuikChange II Site-Directed Mutagenesis kit, with the exception that KOD Hot Start DNA polymerase (Sigma-Aldrich, 71086-3) and NEB Turbo Competent *E. coli* were used. Briefly, the PCR product was amplified with pET28–BilR as the template, using the forward primer GAAGTACACGGAGCCGCTCTGATCGGATCATTC and the reverse primer GAATGATCCGATCAGAGCGGCTCCGTGTACTTC. The rest of the steps were performed as described in the site-directed mutagenesis protocols to yield pET28–BilR–D166A + R167A. Mutations were confirmed by Sanger sequencing.

#### Transformation with pET construct

The construct was transformed into *E. coli* T7 express lysY/Iq competent cells using the manufacturer’s protocol (NEB, C3019). The cells were plated on LB plates with 50 μg ml^−1^ kanamycin to select for successfully transformed colonies. The transformed colonies were verified through Sanger sequencing by Azenta, and plasmids were verified through Plasmidsaurus.

#### Purification and circular dichroism

For protein purification, the *E. coli* T7 express lysY/Iq transformed with pET28–*bilR*(DR166AA) or pET28–*bilR* was grown overnight in LB broth with 50 μg ml^−1^ kanamycin. The inoculum (5 ml) was transferred from the overnight cultures into fresh LB broth with 50 μg ml^−1^ kanamycin and was grown to an optical density of approximately 0.5. Expression of the proteins was induced with 0.4 mM isopropyl β-d-1-thiogalactopyranoside, and the cultures were incubated at 25 °C for 18 h. Cells were centrifuged at 3,000 × *g* and were frozen for 2 h before being resuspended in lysis buffer (20 mM Tris, 0.2 M NaCl, 1 mM phenylmethylsulfonyl fluoride, 16 μg ml^−1^ RNase, 0.5 mg ml^−1^ lysozyme). Samples were then sonicated and centrifuged at 21,130 × *g*. Sodium dodecyl sulfate-polyacrylamide gel electrophoresis was used to verify that the protein was present in the supernatant of the lysate. The supernatant was then filtered using a sterile 0.2 μm filter and purified using a nickel column (G Biosciences nickel-chelating resin) and 5 ml gravity filtration column (G Biosciences columns, 5 ml). The purified proteins were then eluted using filter sterilized elution buffer (20 mM Tris, 0.2 M NaCl, 200 mM imidazole, pH 6.5). Circular dichroism measurements were taken for the purified wild-type BilR protein and mutant BilR(DR166AA) in 20 mM Tris buffer with 50 mM sodium chloride at a pH of 7. Circular dichroism measurements were done using the Jasco J-810 spectropolarimeter with a path length of 1 mm, and then the measurements were normalized and visualized using R.

### Bioinformatic analysis

#### Identification of candidate *bilR* genes

Five genomes from the experimentally confirmed bilirubin reducers and five genomes from closely related confirmed non-reducers were downloaded from NCBI. The assembled genomes for *Clostridium bolteae* CC43_001B and *Clostridium innocuum* 6_1_30 were not available, so the raw sequencing data were downloaded from their corresponding bioprojects. Fastq files for the two species were downloaded from SRA using SRA-Tools (version 2.11.0; https://github.com/ncbi/sra-tools), Trim-Galore (version 0.6.7; https://github.com/ncbi/sra-tools) was used to trim and quality filter the Fastq files, and the genomes were assembled using Spades (version 3.15.5)^42^. The genomes were annotated with Prokka (version 1.14.6)^43^. Orthologer (version 2.7.1) was used to group the predicted protein sequences from each genome into orthogroups using default settings^44^. ECPred (version 1.1) was then used to assign the putative EC numbers to each protein sequence using the default setting^45^. The orthogroups were then subset to keep groups that contained only more than 2 proteins assigned to the oxidoreductase EC number (EC: 1.-.-.-), leaving a total of 389 orthogroups. The taxonomic distribution of the proteins within these oxidoreductase orthogroups was profiled to identify orthogroups that were present in the bilirubin-reducing bacteria and absent in the non-reducing species. Only two orthogroups fit the taxonomic distribution. Of these, one was assigned to EC: 1.17.7.4 (4-hydroxy-3-methylbut-2-enyl diphosphate reductase) and unlikely to be bilirubin reductase; the other one was assigned to EC: 1.-.-.- and was further investigated as a putative bilirubin reductase.

#### Structural prediction and molecular docking

The structures for the putative BilR protein from *Ruminococcus gnavus* CC55_001C and the BilR and BilS proteins from *Clostridium symbiosum* WAL-14163 were predicted using AlphaFold (version 2.2.0)^46^. Putative substrate binding pockets were predicted using Fpocket (version 4.0.2) with default settings^47^. The predicted pockets were visualized using PyMOL (version 2.5.0) and were compared to the substrate binding region of the homologous *E. coli* 2,4-dienoyl-CoA reductase (PDB: 1PS9) to identify three putative substrate binding regions on the BilR structure (http://www.pymol.org/). Structures for bilirubin (PubChem compound identifier 5280352) and flavin mononucleotide (FMN) (PubChem compound identifier 643976) were docked on the *Ruminococcus gnavus* BilR structure using AutoDock Vina (version 1.2.0)^48,49^. The docking simulations were performed within 20 Å × 20 Å × 20 Å cubes centred on the centre points of the three chosen Fpocket substrate binding pocket predictions with the exhaustiveness set to 32. The docking results were visualized using PyMOL, and the results used within figures were chosen based on what is known about what bonds are reduced on bilirubin and based on comparisons to the known binding conformations of the *E. coli* 1PS9 protein. Protein structural alignments were performed between the *Ruminococcus gnavus* CC55_001C predicted structure and each of the *Clostridium symbiosum* WAL-14163 BilR, *Clostridium symbiosum* WAL-14163 BilS and *E. coli* 1PS9 structures using TM-Align^50^. Protein sequence conservation was visualized on the predicted *Ruminococcus gnavus* BilR structure using ConSurf based on the aligned clade 1 BilR sequences (https://consurf.tau.ac.il/consurf_index.php).

#### Search for BilR in the GTDB

The alignment of the amino acid sequences from species in clade 1 (Fig. 4a) and the positions in the alignment corresponding to the first 373 residues of the *Ruminococcus gnavus* CC55_001C BilR protein were extracted to generate a hidden Markov model (HMM) profile using the hmmbuild tool. This profile represents the TIM barrel domain of the BilR proteins. The HMM profile was then used to perform a search within genomes of the GTDB^51^ to profile the taxonomic distribution of BilR. The search was performed using hmmsearch, and only hits below an *e*-value of 1 × 10^−100^ were considered. A protein domain prediction was then performed on the putative BilR hits using InterProScan (version 5.57–90.0)^52^. The putative BilR sequences were filtered requiring that they be at least 50% of the length of the BilR sequences from *Clostridium symbiosum* WAL-14163, that they have the highly conserved HGDR motif present in their sequence and that they have only the expected domains present (PF00724 for the shorter *bilR* or PF07992 and PF00724 for the longer BilR version). The presence or absence of BilR was then summarized across the different bacterial taxa, and the results were visualized using iTOL^53^.

#### Profiling of *bilR* presence in the human gut

A collection of metagenome datasets passing basic quality control was curated to provide a cross section of gut metagenomes from infants in the first year of life (*n* = 4,296), patients with IBD (*n* = 1,863) and healthy adults (*n* = 1,801) (Supplementary Table 2). A *bilR* reference gene dataset was generated by searching the Unified Human Gastrointestinal Genome collection using the previously generated HMM profile of the BilR TIM barrel domain. The resulting hits were filtered based on a 1 × 10^−100^ *e*-value threshold and were subjected to the same quality control used in the search against the GTDB, resulting in a total of 11,158 *bilR* reference sequences. The metagenomes were all processed using a standard workflow that consisted of the following steps: (1) the reads were downloaded from SRA, (2) the adaptors were trimmed using Trim-Galore with default settings, (3) potential human contaminant reads were identified by mapping the reads to a human genome reference (assembly T2T-CHM13v2.0) and were removed using Samtools (version 1.16.1)^54^, (4) samples with less than one million reads were not kept and (5) reads were aligned with the *bilR* reference gene set using Bowtie2 (ref. ^55^). The number of reads mapped to the *bilR* reference database was then summarized for each sample by normalizing by the total number of reads in the sample and multiplying by one million to give a *bilR* counts per million value (CPM).

Infant-related metagenomes were binned into age groups of 30 days from 0 day to 1 year of life, and samples without age metadata were not considered in this analysis. Samples from patients with IBD were categorized based on whether the patient had ulcerative colitis or Crohn’s disease. For healthy gut metagenomes, samples were excluded if they were from patients below 3 years of age. *bilR* was considered to be present in a sample if the *bilR* CPM value was greater than five CPM. The prevalence of *bilR* between groups was compared using a test of equal proportions, using the ‘prop.test’ function from the R ‘stats’ library, to test if the given proportion of samples with *bilR* present was different between two groups.

### Reporting summary

Further information on research design is available in the Nature Portfolio Reporting Summary linked to this article.

### Supplementary information

Reporting Summary

Supplementary Tables Supplementary Table 1: Genomes from GTDB with detected bilirubin reductase genes. Supplementary Table 2: Metadata associated with analysed metagenomic samples.

## Data Availability

Sequences for the *bilR* genes from *Clostridioides difficile*, *Clostridium symbiosum* and *Ruminococcus gnavus* are available in RefSeq under accessions WP_021359617.1, WP_003504328.1 and WP_009244284.1, respectively. All genomic data analysed in this study are available through the GTDB (https://gtdb.ecogenomic.org/) or Unified Human Gastrointestinal Genome collection (https://www.ebi.ac.uk/ena/browser/view/PRJEB33885). All metagenomic datasets analysed in the study are publicly available, and the project and run information is detailed in Supplementary Table 2. The human reference genome used during metagenome analysis (assembly T2T-CHM13v2.0) is available in the NCBI RefSeq database (accession GCF_009914755.1). The data used to generate the figures related to the metabolomics and fluorescence analysis are provided in the GitHub repository: https://github.com/nlm-irp-jianglab/bilirubin-bioinfo.git (10.5281/zenodo.10058858)^56^.

## Code availability

Scripts related to the processing and analysis of the metagenomic data along with scripts and data for the analysis of the metabolomics and fluorescence data are provided in the following GitHub repository: https://github.com/nlm-irp-jianglab/bilirubin-bioinfo.git (10.5281/zenodo.10058858)^56^.

## References

[1] Koizumi S (2007). Human heme oxygenase-1 deficiency: a lesson on serendipity in the discovery of the novel disease. Pediatr. Int..

[2] Lester, R. & Schmid, R. Intestinal absorption of bile pigments—bilirubin absorption in man. *N. Engl. J. Med.*10.1056/nejm196307252690402 (1963). 10.1056/NEJM196307252690402 13929921

[3] Lester R, Schmid R (1965). Intestinal absorption of bile pigments. III. The enterohepatic circulation of urobilinogen in the rat. J. Clin. Invest..

[4] Saxerholt H, Skar V, Midtvedt T (1990). HPLC separation and quantification of bilirubin and its glucuronide conjugates in faeces and intestinal contents of germ-free rats. Scand. J. Clin. Lab. Invest..

[5] With, T. K. *Bile Pigments: Chemical, Biological, and Clinical Aspects* (Academic Press, 1968).

[6] Ching S, Ingram D, Hahnel R, Beilby J, Rossi E (2002). Serum levels of micronutrients, antioxidants and total antioxidant status predict risk of breast cancer in a case control study. J. Nutr..

[7] Osiak W, Wątroba S, Kapka-Skrzypczak L, Kurzepa J (2020). Two faces of heme catabolic pathway in newborns: a potential role of bilirubin and carbon monoxide in neonatal inflammatory diseases. Oxid. Med. Cell. Longev..

[8] Kapitulnik J (2004). Bilirubin: an endogenous product of heme degradation with both cytotoxic and cytoprotective properties. Mol. Pharmacol..

[9] Stenemo M (2019). The metabolites urobilin and sphingomyelin (30:1) are associated with incident heart failure in the general population. ESC Heart Fail..

[10] Kipp ZA (2023). Bilirubin levels are negatively correlated with adiposity in obese men and women, and its catabolized product, urobilin, is positively associated with insulin resistance. Antioxidants.

[11] Vítek L, Zelenka J, Zadinová M, Malina J (2005). The impact of intestinal microflora on serum bilirubin levels. J. Hepatol..

[12] Vítek L (2000). Intestinal colonization leading to fecal urobilinoid excretion may play a role in the pathogenesis of neonatal jaundice. J. Pediatr. Gastroenterol. Nutr..

[13] Poland RL, Odell GB (1971). Physiologic jaundice: the enterohepatic circulation of bilirubin. N. Engl. J. Med..

[14] Brodersen R, Hermann LS (1963). Intestinal reabsorption of unconjugated bilirubin. A possible contributing factor in neonatal jaundice. Lancet.

[15] Gustafsson BE, Lanke LS (1960). Bilirubin and urobilins in germfree, ex-germfree, and conventional rats. J. Exp. Med..

[16] Midtvedt T, Gustafsson BE (1981). Microbial conversion of bilirubin to urobilins in vitro and in vivo. Acta Pathol. Microbiol. Scand. B.

[17] Fahmy K, Gray CH, Nicholson DC (1972). The reduction of bile pigments by faecal and intestinal bacteria. Biochim. Biophys. Acta.

[18] Kumagai A (2013). A bilirubin-inducible fluorescent protein from eel muscle. Cell.

[19] Vítek L (2006). Identification of bilirubin reduction products formed by *Clostridium perfringens* isolated from human neonatal fecal flora. J. Chromatogr. B.

[20] Rekittke I (2008). Structure of (E)-4-hydroxy-3-methyl-but-2-enyl diphosphate reductase, the terminal enzyme of the non-mevalonate pathway. J. Am. Chem. Soc..

[21] Hubbard PA, Liang X, Schulz H, Kim J-JP (2003). The crystal structure and reaction mechanism of *Escherichia coli* 2,4-dienoyl-CoA reductase. J. Biol. Chem..

[22] Tu X, Hubbard PA, Kim J-JP, Schulz H (2008). Two distinct proton donors at the active site of *Escherichia coli* 2,4-dienoyl-CoA reductase are responsible for the formation of different products. Biochemistry.

[23] Dennery PA, Seidman DS, Stevenson DK (2001). Neonatal hyperbilirubinemia. N. Engl. J. Med..

[24] Zhao X (2019). The relationship between serum bilirubin and inflammatory bowel disease. Mediators Inflamm..

[25] Brink MA (1999). Enterohepatic cycling of bilirubin: a putative mechanism for pigment gallstone formation in ileal Crohn’s disease. Gastroenterology.

[26] Lloyd-Price J (2019). Multi-omics of the gut microbial ecosystem in inflammatory bowel diseases. Nature.

[27] Garrod AE (1897). Note on the origin of the yellow pigment of urine. J. Physiol..

[28] Weller SDV (1951). Bile pigments in the stools of infants. Arch. Dis. Child..

[29] Maclagan NF (1946). Faecal urobilinogen: clinical evaluation of a simplified method of estimation. Br. J. Exp. Pathol..

[30] Chen S, Tukey RH (2018). Humanized *UGT1* mice, regulation of *UGT1A1*, and the role of the intestinal tract in neonatal hyperbilirubinemia and breast milk-induced jaundice. Drug Metab. Dispos..

[31] Koníčková R (2012). Reduction of bilirubin ditaurate by the intestinal bacterium *Clostridium perfringens*. Acta Biochim. Pol..

[32] Vítek L, Ostrow JD (2009). Bilirubin chemistry and metabolism; harmful and protective aspects. Curr. Pharm. Des..

[33] Zhou S (2019). Association of serum bilirubin in newborns affected by jaundice with gut microbiota dysbiosis. J. Nutr. Biochem..

[34] Vítek L, Novotný L, Sperl M, Holaj R, Spácil J (2006). The inverse association of elevated serum bilirubin levels with subclinical carotid atherosclerosis. Cerebrovasc. Dis..

[35] The Integrative HMP (iHMP) Research Network Consortium. The integrative human microbiome project. *Nature* **569**, 641–648 (2019). 10.1038/s41586-019-1238-8 PMC6784865 31142853

[36] Naumann HN (1947). Schlesinger’s test for urobilin in the presence of riboflavin and other fluorescent compounds. J. Lab. Clin. Med..

[37] Elman R, McMaster PD (1925). Studies on urobilin physiology and pathology: I. The quantitative determination of urobilin. J. Exp. Med..

[38] Kotal P, Fevery J (1991). Quantitation of urobilinogen in feces, urine, bile and serum by direct spectrophotometry of zinc complex. Clin. Chim. Acta.

[39] Daub, B. *Application of 2D Fluorescence Spectroscopy on Faecal Pigments in Water*. Master’s thesis, Swedish Univ. Agricultural Sciences (2017); https://stud.epsilon.slu.se/10251/1/daub_b_170622.pdf

[40] Miyabara Y, Tabata M, Suzuki J, Suzuki S (1992). Separation and sensitive determination of i-urobilin and 1-stercobilin by high-performance liquid chromatography with fluorimetric detection. J. Chromatogr..

[41] Saris, P. E. J., Paulin, L. G. & Uhlén, M. Direct amplication of DNA from colonies of *Bacillus subtilis* and *Escherichia coli* by the polymerase chain reaction. *J. Microbiol. Methods* **11**, 121–126 (1990).

[42] Prjibelski A, Antipov D, Meleshko D, Lapidus A, Korobeynikov A (2020). Using SPAdes de novo assembler. Curr. Protoc. Bioinform..

[43] Seemann T (2014). Prokka: rapid prokaryotic genome annotation. Bioinformatics.

[44] Kuznetsov D (2023). OrthoDB v11: annotation of orthologs in the widest sampling of organismal diversity. Nucleic Acids Res..

[45] Dalkiran A (2018). ECPred: a tool for the prediction of the enzymatic functions of protein sequences based on the EC nomenclature. BMC Bioinform..

[46] Jumper J (2021). Highly accurate protein structure prediction with AlphaFold. Nature.

[47] Le Guilloux V, Schmidtke P, Tuffery P (2009). Fpocket: an open source platform for ligand pocket detection. BMC Bioinform..

[48] Eberhardt, J., Santos-Martins, D., Tillack, A. & Forli, S. AutoDock Vina 1.2.0: new docking methods, expanded force field, and Python bindings. *J. Chem. Inf. Model* **61**, 3891–3898 (2021). 10.1021/acs.jcim.1c00203 PMC10683950 34278794

[49] Trott O, Olson AJ (2010). AutoDock Vina: improving the speed and accuracy of docking with a new scoring function, efficient optimization, and multithreading. J. Comput. Chem..

[50] Zhang Y, Skolnick J (2005). TM-align: a protein structure alignment algorithm based on the TM-score. Nucleic Acids Res..

[51] Parks DH (2022). GTDB: an ongoing census of bacterial and archaeal diversity through a phylogenetically consistent, rank normalized and complete genome-based taxonomy. Nucleic Acids Res..

[52] Jones P (2014). InterProScan 5: genome-scale protein function classification. Bioinformatics.

[53] Letunic I, Bork P (2021). Interactive Tree Of Life (iTOL) v5: an online tool for phylogenetic tree display and annotation. Nucleic Acids Res..

[54] Danecek P (2021). Twelve years of SAMtools and BCFtools. Gigascience.

[55] Langmead B, Salzberg SL (2012). Fast gapped-read alignment with Bowtie 2. Nat. Methods.

[56] Dufault-Thompson, K. nlm-irp-jianglab/bilirubin-bioinfo: initial publication release. *Zenodo*10.5281/zenodo.10058858 (2023).

