# Supplementary material for: BilR is a gut microbial enzyme that reduces bilirubin to urobilinogen
Source: Nat Microbiol. 2024 Jan 3;9(1):173–84. doi: 10.1038/s41564-023-01549-x (PMC10769871; doi:10.1038/s41564-023-01549-x)
Supplement: Supplementary file 1 — Reporting Summary [file 41564_2023_1549_MOESM1_ESM.pdf]

nature portfolio

Corresponding author(s): Brantley Hall, Xiaofang Jiang

Last updated by author(s): Oct 23, 2023

Reporting Summary

Nature Portfolio wishes to improve the reproducibility of the work that we publish. This form provides structure for consistency and transparency in reporting. For further information on Nature Portfolio policies, see our Editorial Policies and the Editorial Policy Checklist.

Statistics

For all statistical analyses, confirm that the following items are present in the figure legend, table legend, main text, or Methods section.

n/a	Confirmed	
☐	☒	The exact sample size (*n*) for each experimental group/condition, given as a discrete number and unit of measurement
☐	☒	A statement on whether measurements were taken from distinct samples or whether the same sample was measured repeatedly
☐	☒	The statistical test(s) used AND whether they are one- or two-sided<br>*Only common tests should be described solely by name; describe more complex techniques in the Methods section.*
☒	☐	A description of all covariates tested
☒	☐	A description of any assumptions or corrections, such as tests of normality and adjustment for multiple comparisons
☐	☒	A full description of the statistical parameters including central tendency (e.g. means) or other basic estimates (e.g. regression coefficient) AND variation (e.g. standard deviation) or associated estimates of uncertainty (e.g. confidence intervals)
☐	☒	For null hypothesis testing, the test statistic (e.g. *F*, *t*, *r*) with confidence intervals, effect sizes, degrees of freedom and *P* value noted<br>*Give P values as exact values whenever suitable.*
☒	☐	For Bayesian analysis, information on the choice of priors and Markov chain Monte Carlo settings
☒	☐	For hierarchical and complex designs, identification of the appropriate level for tests and full reporting of outcomes
☒	☐	Estimates of effect sizes (e.g. Cohen's *d*, Pearson's *r*), indicating how they were calculated

Our web collection on statistics for biologists contains articles on many of the points above.

Software and code

Policy information about availability of computer code

Data collection	Scripts related to the processing and analysis of the metagenomic data along with scripts and data for the analysis of the metabolomics and fluorescence data are provided in the following git repository: https://github.com/nlm-irp-jianglab/bilirubin-bioinfo.git (doi: https://zenodo.org/doi/10.5281/zenodo.10058858) 56
Data analysis	Code used to analyze the metagenomic data and fluorescence/metabolomics data from this study is provided in the https://github.com/nlmirp-jianglab/bilirubin-analysis github repository. In addition multiple other programs were used in these analyses and are listed in the methods section of the manuscript. These include SRA-Toolkit (v. 2.11.0), trim-galore (v. 0.607), Spades (v. 3.15.5), Prokka (v. 1.14.6), Orthologer (v. 2.7.1), EXPred (v 1.1), AlphaFold (v. 2.2.0), Fpocket (v. 4.0.2), PyMOL (v. 2.5.0), AutoDock Vina (v. 1.2.0), Clustal Omega (v. 1.2.4), IQ-TREE (v. 1.2.4), GRASP (v. 30-July-2022), hmmpress (v, 3.3.2), hmmscan (v, 3.3.2), InterProScan (v. 5.57-90.0), Bowtie2 (v 2.5.1), iTol (v 6), and Samtools (v 1.16.1).

For manuscripts utilizing custom algorithms or software that are central to the research but not yet described in published literature, software must be made available to editors and reviewers. We strongly encourage code deposition in a community repository (e.g. GitHub). See the Nature Portfolio guidelines for submitting code & software for further information.

Data

Policy information about availability of data

All manuscripts must include a data availability statement. This statement should provide the following information, where applicable:

- Accession codes, unique identifiers, or web links for publicly available datasets
- A description of any restrictions on data availability
- For clinical datasets or third party data, please ensure that the statement adheres to our policy

Sequences for the bilR genes from C. difficile , C. symbiosum, and R. gnavus are available in RefSeq under accessions WP_021359617.1, WP_003504328.1, and WP_009244284.1 respectively. All genomic data analyzed in this study are available through the Genome Taxonomy Database (https://gtdb.ecogenomic.org/) or Unified Human Gastrointestinal Genome collection (https://www.ebi.ac.uk/ena/browser/view/PRJEB33885). All metagenomic datasets analyzed in the study are publicly available and the project and run information is detailed in Supplementary Table 2. The human reference genome used during metagenome analysis (assembly T2T-CHM13v2.0) is available in the NCBI RefSeq database (accession GCF_009914755.1). The data used to generate the figures related to the metabolomics and fluorescence analysis is provided in the github repository: https://github.com/nlm-irp-jianglab/bilirubin-bioinfo.git (doi: https://zenodo.org/doi/10.5281/zenodo.10058858) 56

Research involving human participants, their data, or biological material

Policy information about studies with human participants or human data. See also policy information about sex, gender (identity/presentation), and sexual orientation and race, ethnicity and racism.

Reporting on sex and gender	Not Applicable
Reporting on race, ethnicity, or other socially relevant groupings	Not Applicable
Population characteristics	Not Applicable
Recruitment	Not Applicable
Ethics oversight	Not Applicable

Note that full information on the approval of the study protocol must also be provided in the manuscript.

Field-specific reporting

Please select the one below that is the best fit for your research. If you are not sure, read the appropriate sections before making your selection.

☒ Life sciences ☐ Behavioural & social sciences ☐ Ecological, evolutionary & environmental sciences

For a reference copy of the document with all sections, see nature.com/documents/nr-reporting-summary-flat.pdf

Life sciences study design

All studies must disclose on these points even when the disclosure is negative.

Sample size	Biological triplicates or quadruplicates were used for bilirubin reduction experiments to obtain a good estimate of the bacterial strains' abilities to reduce bilirubin. The sample sizes used were based on the commonly used standards for microbiology experiments. Due to resource limitations some strains were only able to be screened using single biological replicates, these are noted in the manuscript.
Data exclusions	No data was excluded from the analyses.
Replication	Fluorescence assays were validated through metabolomics when possible to verify that the fluorescence assay developed in this study reliably detected bilirubin and not other bilin metabolites. Experiments were typically performed in biological triplicate or quadruplicate, with the experiments related to the mutant R. gnavus bilR transformed strains being repeated with six biological replicates.<br>Due to resource limitations the fluorescence screening for bilirubin reduction for strains Clostridium sp. M62/1 and C. citroniae WAL-17108 were only performed as single biological replicates.
Randomization	Randomization is not relevant to the experiments being performed in this study.
Blinding	Blinding is not relevant to the experiments being performed in this study.

Reporting for specific materials, systems and methods

We require information from authors about some types of materials, experimental systems and methods used in many studies. Here, indicate whether each material, system or method listed is relevant to your study. If you are not sure if a list item applies to your research, read the appropriate section before selecting a response.

Materials & experimental systems

n/a	Involved in the study
☒	☐ Antibodies
☒	☐ Eukaryotic cell lines
☒	☐ Palaeontology and archaeology
☒	☐ Animals and other organisms
☒	☐ Clinical data
☒	☐ Dual use research of concern
☒	☐ Plants

Methods

n/a	Involved in the study
☒	☐ ChIP-seq
☒	☐ Flow cytometry
☒	☐ MRI-based neuroimaging

Plants

Seed stocks	No plants were used in this study.
Novel plant genotypes	No plants were used in this study.
Authentication	No plants were used in this study.
